# Genetic linkage map of willow (*Salix leucopithecia×S.erioclada L*) based on AFLP and SSR markers

**DOI:** 10.1186/1753-6561-5-S7-P29

**Published:** 2011-09-13

**Authors:** Jian-Jun Hu, Jin-Hui Lv, Meng-Zhu Lu

**Affiliations:** 1 Research Institute of Forestry, Chinese Academy of Forestry, Beijing 100091, PR China

## 

Due to its fast growth rate and little nursery requirements, willow is largely planted as short rotation coppice species for bioenergy resources in temperate regions. Willow coppices can be easily established by vegetative propagation of cuttings and can remain productive for up to 25 years. In China, diploid species *S. leucopithecia* and *S. erioclada* L. are two promising willow species that can be used for high biomass plantation. In order to further improve the biomass yield and resistance to drought and pests, we tried to create a genetic map to assist willow breeding. 560 F_1_ individuals from a cross between *S. leucopithecia* x *S. erioclada* L were used to generate a willow genetic map using AFLP and SSR markers.

Both *Populus* and *Salix* are members of the Salicaceae. They share genomic homologues with high similarity and many common biological traits. As a model species for biological studies in trees,*Populus* has considerable genetic and genomic resources, which could provide cues for willow study. Five hundred pairs of SSR primers were selected from *Populus* database for willow analysis; 88 of them successfully generated polymorphic loci between *S. leucopithecia* x *S. erioclada* L. In addition, 24 pairs of AFLP primers also succeeded in detecting polymorphic fragments from F_1_ individuals.

We selected 243 individuals to construct a framework linkage maps of parents using a two-way pseudo-testcross strategy. The linkage map of *S.erioclada* L contains 98 loci, organized in 15 linkage groups with total map distances between 76.7 cM and 209.5 cM. The average map distance between markers is 18.6 cM and the total genetic distance is about 1,810 cM, covering estimated 77.13% of the genome. The linkage map of *S. leucopithecia* contains 60 loci, arranged into 9 groups with map distances between 63.5 cM and 149.1 cM. The average map distance between markers is 19.57 cM, and the total genetic distance is about 1,037 cM, covering estimated 70.3% of the genome.

